# Sudangrass, an alternative lignocellulosic feedstock for bioenergy in Argentina

**DOI:** 10.1371/journal.pone.0217435

**Published:** 2019-05-23

**Authors:** Alberto Acevedo, Rachael Simister, Simon J. McQueen-Mason, Leonardo D. Gómez

**Affiliations:** 1 Instituto de Suelos, Centro de Investigación de Recursos Naturales, Castelar, Buenos Aires, Argentina; 2 Instituto Nacional de Tecnología Agropecuaria, Castelar, Buenos Aires, Argentina; 3 Centre for Novel Agricultural Products, Department of Biology, University of York, York, United Kingdom; University of Sao Paulo, BRAZIL

## Abstract

Sudangrass, *Sorghum sudanense* (Piper) Stapf, is a vigorous forage crop that has also been used for biogas, paper, and electricity production. Due to the large biomass yields achieved by sudangrass and the large area of potential growth in Argentina seven sudangrass accessions from a collection of *S*. *sudanense* were analyzed to evaluate their potential as feedstocks for lignocellulosic bioethanol production, and to assess whether there is an association between the response to biotic and abiotic stresses and the composition of the biomass. The biomass composition was analyzed for major cell wall polymers, monosaccharides, and elemental composition. On average, 68% of stem lignocellulosic biomass was comprised of matrix polysaccharides and crystalline cellulose, representing a potential source of sugars for bioethanol production. Xylose was the predominant matrix polysaccharide monosaccharide comprising, on average, 45% of the total sugars, followed by arabinose, glucose, galactose, galacturonic acid, mannose, glucuronic acid, and fucose. Rhamnose was not detected in any of the biomasses analyzed. Silica was the most abundant element in sudangrass stem, followed by chloride, calcium, phosphorus and sulfur. We performed saccharification analyses after pretreatments. Alkaline pretreatment was more effective than water pretreatment. Sodium hydroxide pretreatment exposed different levels of recalcitrance among sudangrass accessions, whereas the water pretreatment did not. Phenological traits were also evaluated, showing significant variability among accessions. The comparison of major cell wall polymers and monosaccharide composition between tolerant and susceptible accessions to abiotic and biotic stresses suggests an association between the composition of the biomass and the response to stress.

## Introduction

Argentina is a large country (3.761.274 km^2^) and has a range of different climates and soils. Despite this environmental diversity, only three crops stand as feedstocks for biofuel production in the country. Corn and sugarcane are used as feedstocks for first generation (1G) bioethanol production and soybean is used as feedstock for biodiesel production. Together they account for more than 90% of both the productivity and the planted area estimated for 2019 [1]. Independently of the market to which these crops are destined for, it would be highly desirable to increase the diversity of feedstocks in terms of the productivity and planted area. Argentina has a number of industrial plants that produce 1G bioethanol but currently there is no production of second generation (2G) bioethanol. Given the biomass production in the country, there is a possibility of establishing 2G bioethanol production. Besides policy, investment and demand/supply chains, one important issue in this area is the prospection of suitable biomass feedstock.

The *Sorghum* genus includes three distinct morphotypes that are used as forages: sudangrass, forage sorghums, and sorghum x sudangrass hybrids [2]. Sudangrass is a vigorous forage crop that has a remarkable drought tolerance [3], making it an ideal alternative to fill feed gaps during water shortages. As a C4 species and summer cover crop, it also has the potential to produce large amounts of biomass that will build soil quality in a short period of time, contributing or recycling nitrogen, outcompeting weed growth and reducing soil erosion by keeping the soil surface covered [4]. However, as a warm-season crop plants are greatly injured or even killed by frost [5]. In Argentina, sudangrass used as green feed, does not cover the main feed deficit in winter. However, in summer it allows the concentration of many cattle heads per unit area, and with good management can help achieving higher weight gains than with natural grasslands [6, 7]. Besides these advantages, it has also been used as feedstock for biogas, paper, and electricity production [8].

Due to the large biomass yields achieved by sudangrass and the large area of potential growth in Argentina, we propose here that it could constitute a suitable feedstock for 2G ethanol production. Its development as a feedstock would require a deeper knowledge of the biomass composition and variability across genotypes in their stress responses to an increasingly unpredictable environment. In this study, phenological and agricultural parameters, as well as the biomass composition in stems of sudangrass accessions have been investigated in order to evaluate the potential of this species as feedstock for producing advanced biofuels. The association between the composition of the biomass and the response to biotic and abiotic stresses, under different environments, has also been assessed.

## Materials and methods

### Plant material

Plants of different accessions of sudangrass (*Sorghum sudanense* (Piper) Stapf) [9] obtained from the germplasm collection of sorghum of the National Institute of Agricultural Technology, INTA (Argentina) were field-grown in 2012–2013 at the Manfredi Research Experimental Station, Manfredi, Córdoba, Argentina.

Seven *S*. *sudanense* accessions (*R*.*S 2199 Sudanense*, *R*.*S 2198 Sudanense*, *R*.*S 1370 Syn4*, *R*.*S 841 Sudanense*, *R*.*S 1601 Tift*, *R*.*S 1594 Wealer*, and *R*.*S 1731 Juar 20*) were randomly chosen from the collection and the 5^th^ internodes of 10 plants from each accession were used for determining the composition of cell wall components. The 5^th^ internodes were harvested at plant senescence and the 10 samples from each accession were pooled from which three replicates by sample were analyzed.

#### Response to stresses

Response to spontaneous infection of bacterioses and stem borer attack and to spontaneous occurrence of drought and frost was measured throughout plant life cycle in the seven field-grown sudangrass accessions as plants encountered these stresses sometime during their growth period. Response to the named stresses was measured upon a scale that ranged from 0 to 4, where 0 stands for resistance and 4 for susceptibility [10]. Additional sudangrass accessions (*R*.*S 1842 Honey Sor*. for bacterioses and stem borer, and *R*.*S 2305 I*.*S*. *1143 N*.*12* for drought and frost) were also grown at Manfredi´s research experimental field and used as susceptibility controls because they have shown the same response to these stresses every time INTA´s sorghum germplasm collection was grown in the field. Therefore, these susceptibility controls were used instead of control groups for each of the seven genotypes that were not exposed to these stresses.

Bacterioses and stem borer attack caused damage in leaf-blade and bottom and middle part of stems, respectively. Drought and frost, defined as temperature below the basal temperature of sorghum, primarily caused flower abortion.

Climatic data (average, maximum and minimum temperature, rainfall, frost, average humidity, average and maximum wind speed, and soil temperature) were automatically registered on a daily basis and throughout plant life cycle by a meteorological station located at the Manfredi Research Experimental Station (S1 Table). The field was not irrigated, thus water provision to plants depended on rainfall.

#### Phenological traits

Phenological traits were measured for the 7 different accessions (7 plants per accession). Plant height, panicle exertion, and tiller number were measured at the time at which 50% of the plants of each accession finished growing. Flowering time was determined at the time at which 50% of the plants of each accession had flowered. Days to maturity were determined at the time at which 50% of the plants of each accession had reached maturity developmental stage.

#### Solubles

The pooled fifth internodes of sudangrass stems were milled and the powder was washed with 1.5 ml ethanol, vortexed and spun down for 20 minutes at 13,000 rpm. The supernatant was removed, air dried, washed with 1 ml of 90% aqueous DMSO and left rocking over-night. Then, the sample was centrifuged for 20 minutes, washed 4 times with 1 ml ethanol, and centrifuged for 10 minutes. The samples were dried under vacuum for 90 minutes at 60°C and weighed out after extraction. Finally, the solubles were calculated as the difference between the original sample weight and the washed sample weight, expressed as a percentage.

#### Lignin determination

Biomass powder was weighed out (4 mg) into 2 ml tubes. Lignin was determined according to Foster et al. (2010) [11]. The biomass was heated at 50°C for 3 hours, after adding 250 μl of acetyl bromide solution (25% of acetyl bromide and 75% of glacial acetic acid in volume), vortexing every 15 minutes. After the samples were cooled to room temperature, the content was transferred into 5 ml volumetric flasks. A further 1 ml sodium hydroxide 2 M was used to rinse the tubes, pouring the NaOH into the 5 ml flasks. 175 μl of hydroxylamine HCl 0.5 M was added to the volumetric flasks and, after vortexing, the latter were filled up to 5 ml with glacial acetic acid and mixed several times. Finally, in order to measure the 280 nm UV adsorption by spectrophotometer, 100 μl of each sample was diluted in 900 μl of glacial acetic acid. The amount of lignin was calculated using the following formula: [absorbance/(coefficient pathlength)] · [(total volume · 100%)/biomass weight], where coefficient = 17.75, pathlength = 1, total volume = 5, biomass weight = 4.

#### Matrix polysaccharides

Dry biomass powder (4 mg) was partially hydrolyzed by adding 0.5 ml of 2M TriFluoroAcetic acid. Then, the vials were flushed with dry argon, mixed and heated at 100°C for 4 hours, vortexing periodically. The vials were then cooled to room temperature and dried in centrifugal evaporator with fume extraction overnight. The pellets were washed twice with 500 μl of 2-propanol and vacuum dried. Finally, the samples were resuspended in 200 μl of deionised water, filtered with 0.45 μm PTFE filters, and the monosaccharide profile analyzed. To resolve the monosaccharide profile of non-cellulosic polysaccharides, samples were analyzed using high-performance anion-exchange chromatography (HPAEC) on a CarboPac PA-20 column with pulsed amperometric detection as described in Jones et al. (2003) [12]. Separated monosaccharides were quantified by external calibration using an equimolar mixture of monosaccharide standards, which had also been treated with 2M TFA in the same way.

#### Cellulose

Biomass dry pellets after TFA hydrolysis were washed once with 1.5 ml of water, and three times using 1.5 ml of acetone. The pellets were left to air dry overnight before complete hydrolysis by, adding 90 μl of 72% (p/v) sulfuric acid, incubating at room temperature for 4 hours, 1.89 ml of water was subsequently added and the sample was heated for 4 hours at 120°C. The glucose (Glu) content of the supernatant was assessed using the colorimetric Anthrone assay, using a Glu standard curve.

#### Saccharification analysis

Saccharification potential was determined using an automated robotic platform according to Gomez et al. (2010) [13]. In brief, loading of plant powder into 96-well plates was done using a custom-made robotic platform (Labman Automation, Stokesley, North Yorkshire, UK), and saccharification assays were performed after alkali pretreatment at 94°C for 30 min. Enzymatic hydrolysis was carried out using an enzyme cocktail with a 4:1 ratio of Celluclast and Novozyme 188 (Novozymes Enzymes).

#### Statistical analyses

InfoStat software, version updated 07-10-2018, was used for Statistical analyses [14]. Variance analysis was used to test phenotypic data and cell wall components. Saccharification analyses were tested using general and mixed linear models. LSD Fisher's test was used to compare means.

## Results

### Sudangrass biomass composition

**Fig 1:**
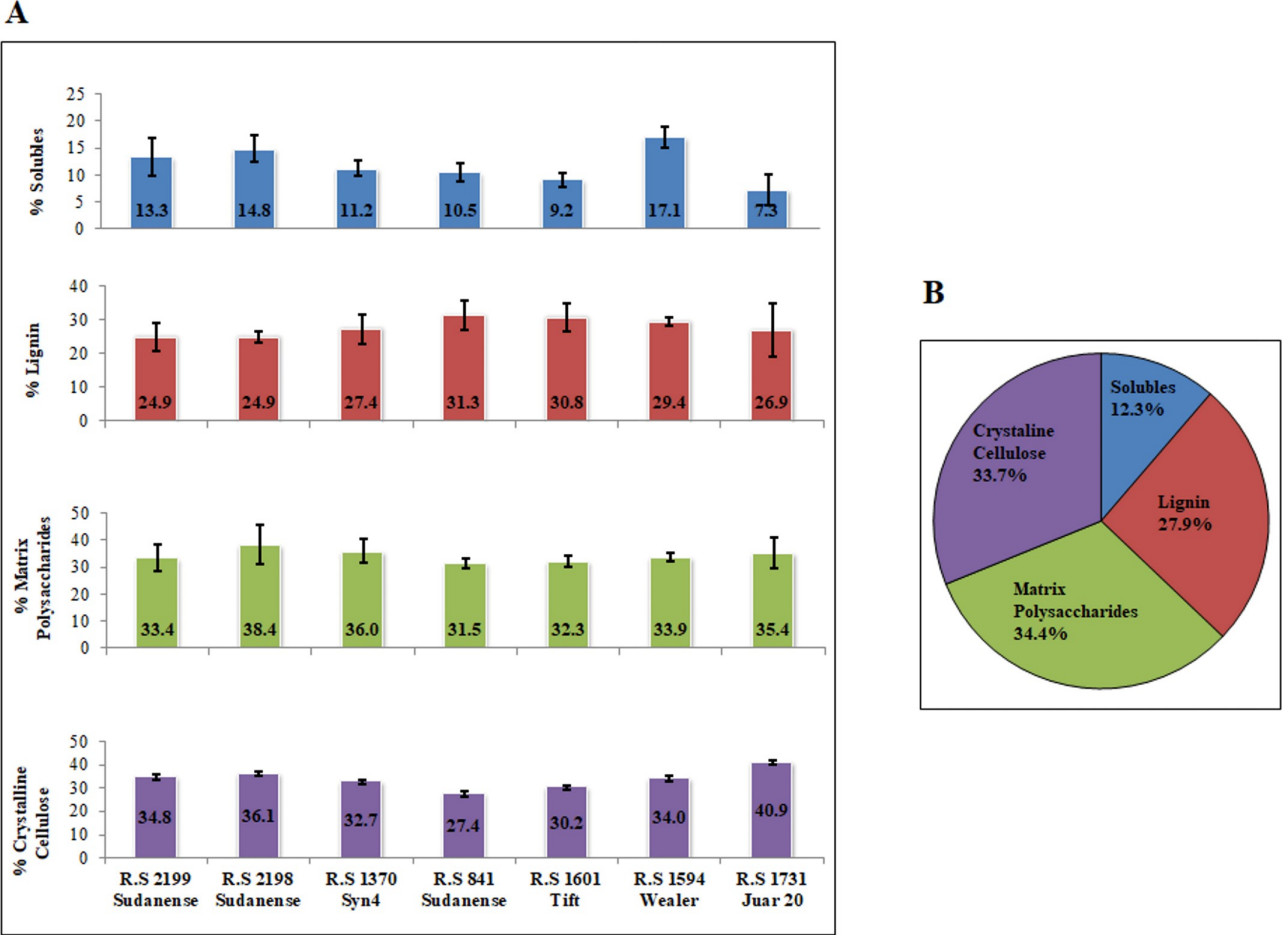
Major cell wall components in stems of sudangrass. (A) Major cell wall components by genotype. Vertical bars stand for standard deviation of the means. Note that soluble extractives show statistically significant differences across accessions (See S2 Table). (B) Average of major cell wall components.

The biomass composition of a feedstock is a key parameter to evaluate the potential of different genotypes for different applications. The major cell wall components were determined in the seven field-grown sudangrass genotypes to evaluate the variability of biomass composition between accessions and, in a longer term, the potential of this species as feedstock for producing cellulosic biofuels. For all seven accessions, the biomass composition was analyzed for soluble extractives, lignin, matrix polysaccharides, and crystalline cellulose contents (Fig 1).

The content of soluble extractives showed statistically significant differences across sudangrass accessions (p = 0.0007) (Fig 1A). Three genotypes (*R*.*S 1594 Wealer*, *R*.*S 2198 Sudanense*, and *R*.*S 2199 Sudanense*) accounted for the highest contents of soluble extractives as compared to the remaining genotypes. In its turn *R*.*S 1731 Juar 20* and R.S 1601 *Tift* showed the lowest contents of soluble extractives and differed significantly from the rest of the accessions (Fig 1A). The percentage of lignin ranged from 31.28% in *R*.*S 841 Sudanense* to 24.94% in *R*.*S 2199* and *R*.*S 2198 Sudanense*, and showed no statistically significant differences across sudangrass accessions (p = 0.5782) (Fig 1A). On average, the matrix polysaccharide fraction (34.42%) was slightly higher than the crystalline cellulose fraction (33.74%) (Fig 1B). The former fraction showed no statistically significant differences across accessions (p = 0.5565) and ranged from 38.44% in *R*.*S 2198 Sudanense* to 31.48% in *R*.*S 841 Sudanense*. The content of crystalline cellulose that ranged from 40.93% in *R*.*S 1731 Juar 20* to 27.44% in *R*.*S 841 Sudanense* also showed no statistically significant differences across accessions (p = 0.5969) (Fig 1A). Taken together, these findings indicate that all the extreme values of the variables assayed were concentrated in only four genotypes *R*.*S 2198 Sudanense*, *R*.*S 841 Sudanense*, *R*.*S 1594 Wealer*, and *R*.*S 1731 Juar 20* (Fig 1A).

**Fig 2:**
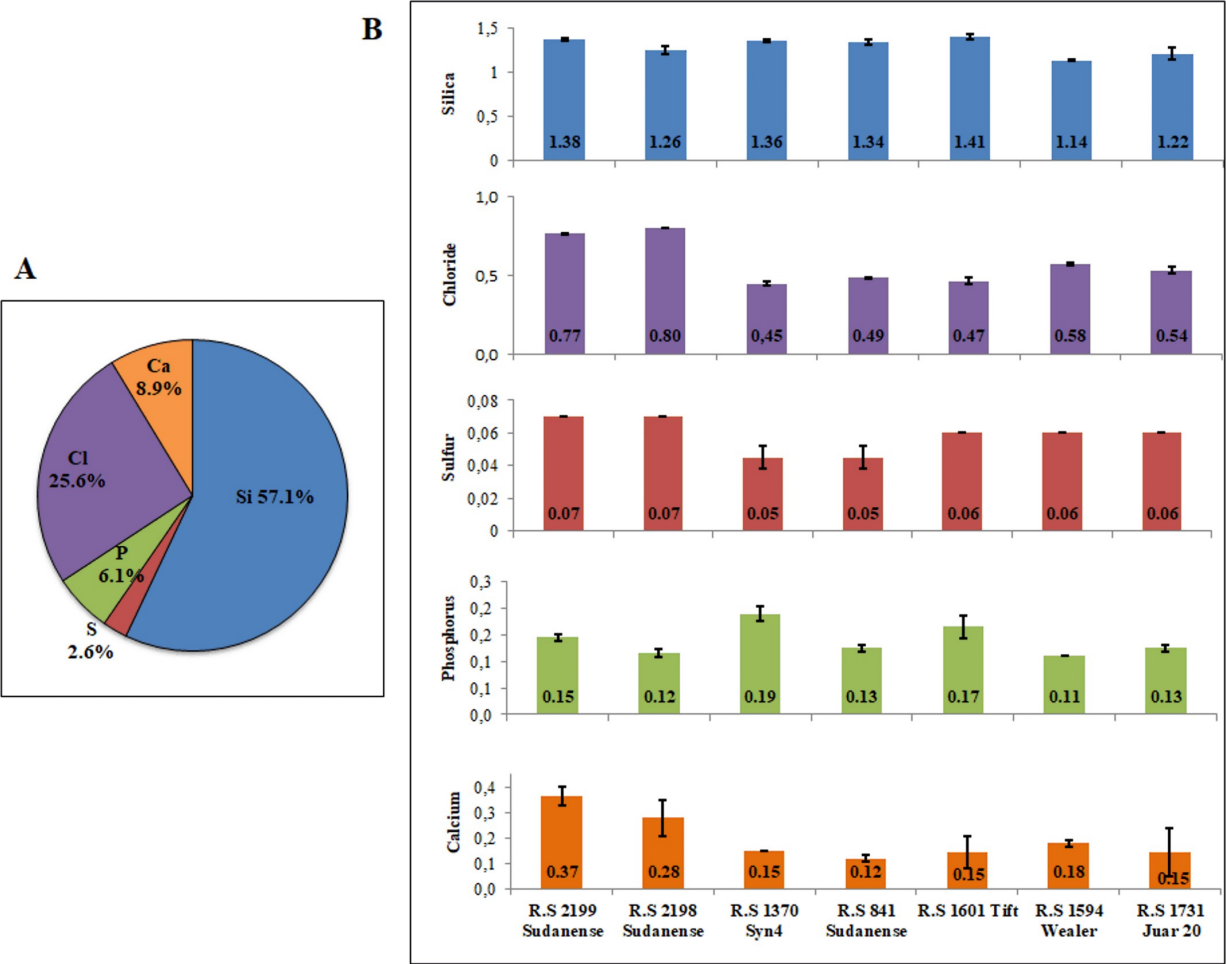
Cell wall elements in stems of sudangrass. (A) Average of elements in cell walls. (B) Cell wall elements by genotype. Vertical bars stand for standard deviation of the means. Note that all the elements analyzed show statistically significant differences across accessions.

The elemental composition of the different biomass samples was determined across accessions (Fig 2). Silica (Si) was the most abundant of the elements determined in sudangrass stem, followed by chloride (Cl), calcium (Ca), phosphorus (P) and sulfur (S) (Fig 2A). Si content showed statistically significant differences across accessions (p = 0.0012) and varied from 1.405% in *R*.*S 1601 Tift* to 1.140% in *R*.*S 1594 Wealer*. Cl content showed statistically significant differences across accessions (p = 0.0001) and varied from 0.800% in *R*.*S 2198 Sudanense* to 0.450% in *R*.*S 1370 Syn4*. Levels of S ranged from 0.070% in *R*.*S 2199 Sudanense* and *R*.*S 2198 Sudanense* to 0.045% in *R*.*S 1370 Syn4* and *R*.*S 841 Sudanense*, and showed statistically significant differences across accessions (p = 0.0012). The variation of P extended from 0.190% in *R*.*S 1370 Syn4* to 0.110% in *R*.*S 1594 Wealer*, and showed statistically significant differences across accessions (p = 0.0014). Ca content showed statistically significant differences across accessions (p = 0.0167). Ca content was highest (0.365%) in *R*.*S 2199 Sudanense*, contrasting with the lowest value (0.120%; 0,145%; 0,145%; 0.150%; and 0.180%) determined in *R*.*S 841 Sudanense*, *R*.*S 1601 Tift*, *R*.*S 1731 Juar 20*, *R*.*S 1370 Syn4*, and *R*.*S 1594 Wealer*, respectively (Fig 2B). Together, these results point out that the content of Si, S, P and Cl varied 27%, 56%, 73% and 78%, respectively in the accessions analyzed; while the variation was 304% for Ca. Moreover, three genotypes contained the lowest values of these elements; whereas, four genotypes were responsible for the uppermost values (Fig 2B).

**Fig 3:**
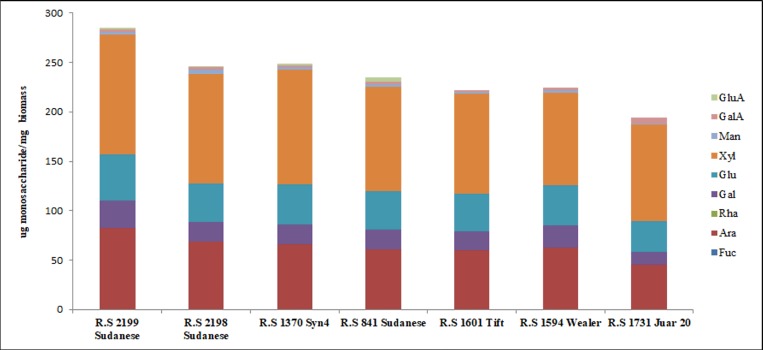
Monosaccharide composition of the matrix polysaccharides in stem cell walls of sudangrass. Monosaccharide composition of the matrix polysaccharides by sudangrass genotype. Note that GalA, Xyl, Gal, and Ara show statistically significant differences across accessions. (S3 Table).

The monosaccharides composition of the matrix polysaccharides of sudangrass stem is shown in Fig 3. Xylose (Xyl) was the predominant monosaccharide comprising, on average, 45.02% of the total sugars, followed by arabinose (Ara) (26.90%), Glu (16.65%), galactose (Gal) (8.50%), galacturonic acid (GalA) (1.26%), mannose (Man) (1.05%), glucuronic acid (GluA) (0.59%), and fucose (Fuc) (0.03%). Rhamnose was not detected in any of the biomasses analyzed (Fig 3). Concentrations of Xyl (p = 0.0003), Ara (p = 0.0084), Gal (p = 0.0503), and GalA (p = 0.0001) showed statistically significant differences across accessions, respectively. *R*.*S 2199 Sudanense* accounted for the highest concentration of the four most abundant monosaccharides: Ara, Gal, Glu, and Xyl; on the other hand, *R*.*S 1731 Juar 20* showed the lowest concentration of Ara, Gal, Glu, and Man (Fig 3B). *R*.*S 2198 Sudanense*, *R*.*S 1731 Juar 20*, and *R*.*S 841 Sudanense* had more Man, GalA, and GluA, as concentrations of these monosaccharides were 1.7-, 2.1-, and 1.8-fold their respective average concentrations (Fig 3B). No GluA was detected in *R*.*S 1601 Tift*, *R*.*S 1594 Wealer*, and *R*.*S 1731 Juar 20* (Fig 3B).

For all seven accessions, the matrix polysaccharide average concentration of pentoses totalled 170.23 μg/mg biomass whereas that of hexoses (excluding sugar acids) reached 62.09 μg/mg sudangrass biomass. The ratio pentoses:hexoses was 2.7:1 in the matrix polysaccharides.

**Fig 4:**
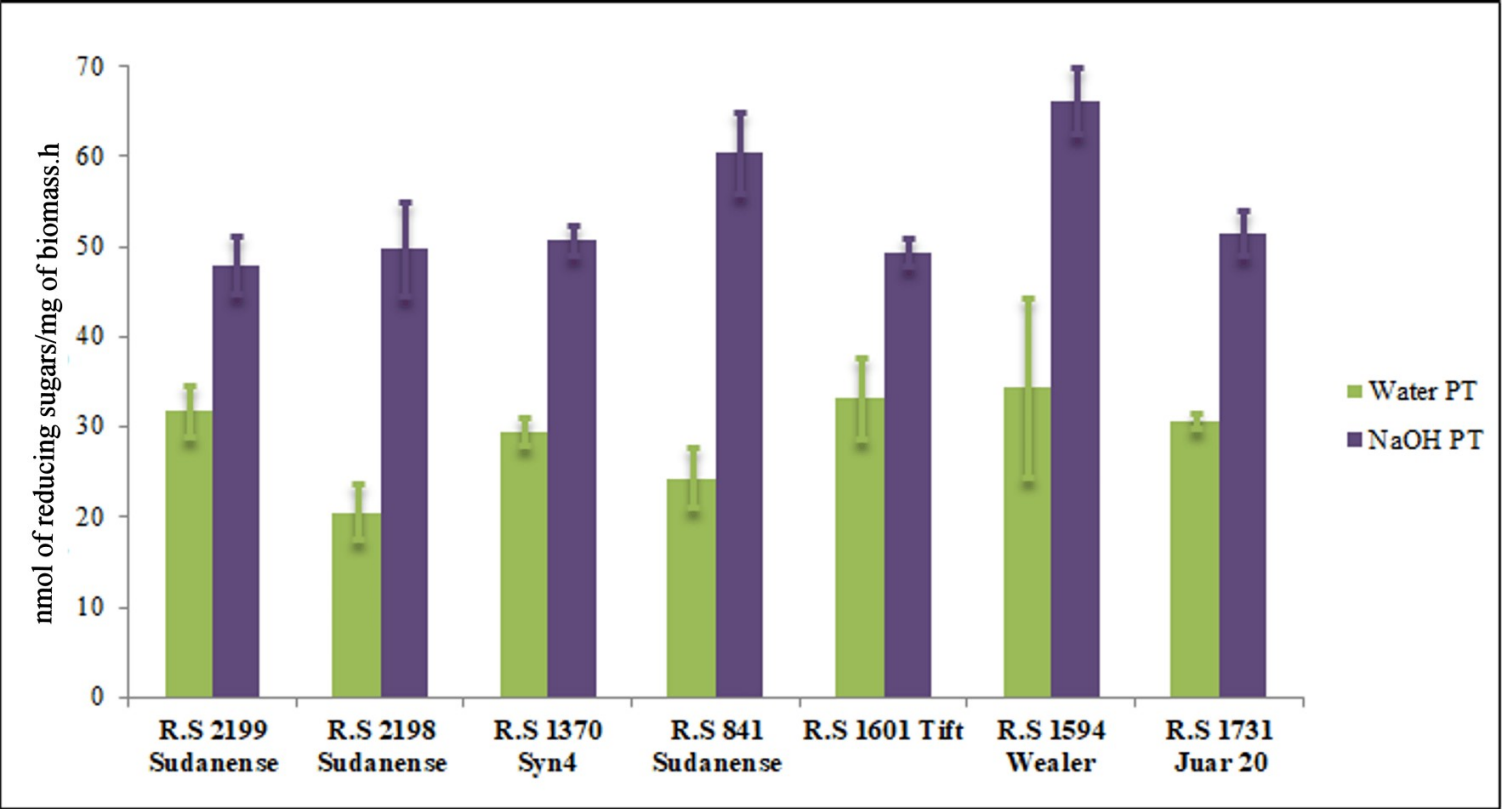
Saccharification analyses across sudangrass accessions following water and sodium hydroxide pretreatments. Vertical bars stand for standard deviation of the means. Note that the sodium hydroxide pretreatment was more effective since more sugars were released than in the water pretreatment. (S4 Table).

Saccharification analyses were performed across accessions following water and sodium hydroxide pretreatments (Fig 4). Statistically significant differences were detected between pretreatments (p <0.0001), across accessions (p <0.0001), and in the pretreatment: accession interaction (p <0.0001). The sodium hydroxide pretreatment was more effective since more sugars were released than in the water pretreatment. The average level of reducing sugars released in *R*.*S 1594 Wealer* and *R*.*S 841 Sudanense* upon sodium hydroxide pretreatment was statistically higher than in the five remaining accessions analyzed, indicating that, under these experimental conditions, these five accessions were far more recalcitrant genotypes than *R*.*S 1594 Wealer* and *R*.*S 841 Sudanense* (Fig 4). Only two genotypes, *R*.*S 1594 Wealer* and *R*.*S 841 Sudanense*, exhibited average levels of released reducing sugars that exceeded the overall average level of reducing sugars (53.63 nmol/mg.h) released across the 7 genotypes after sodium hydroxide pretreatment.

### Phenotypic data and differential response to biotic and abiotic stresses

**Fig 5:**
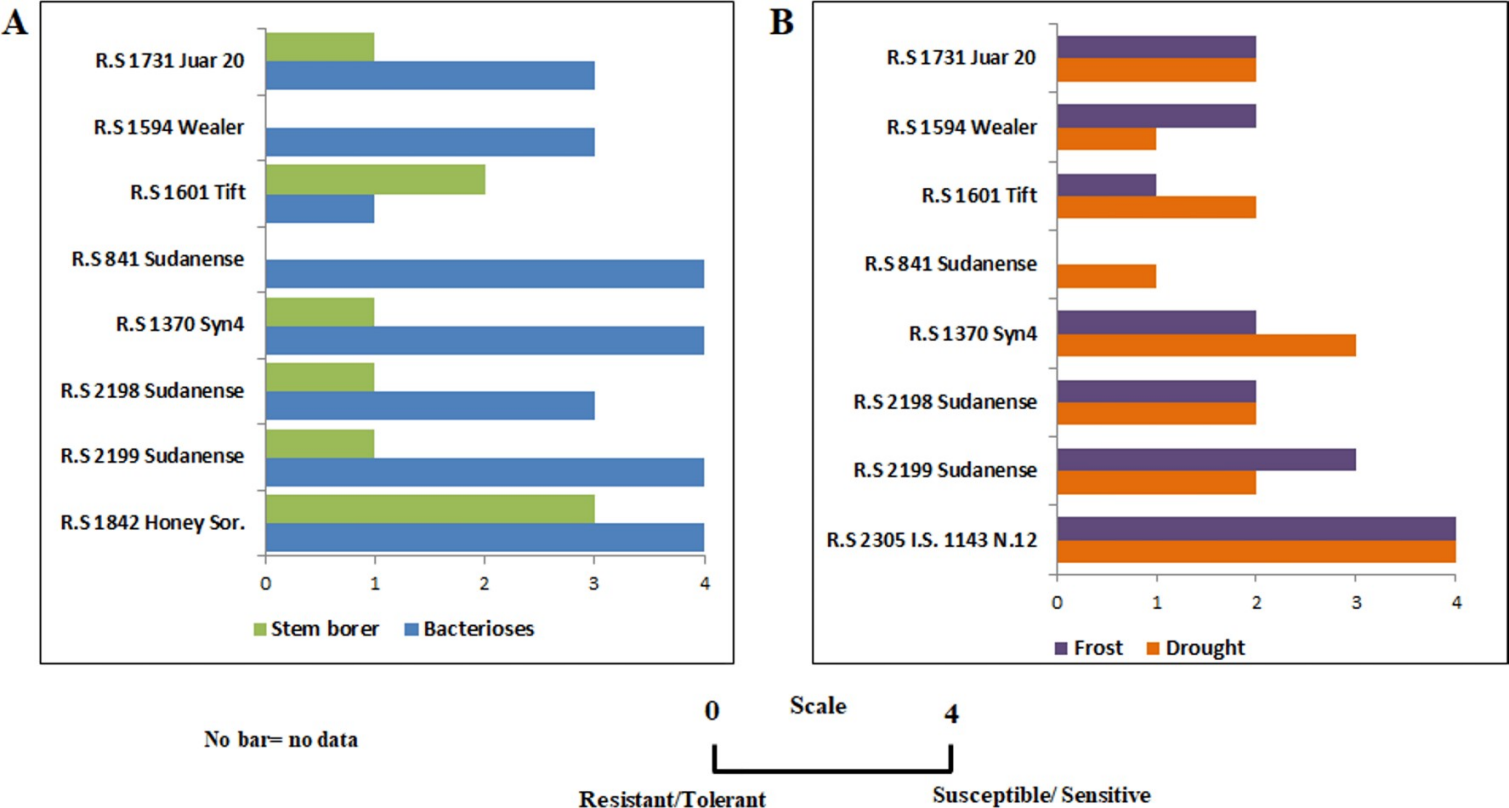
Differential response to biotic and abiotic stresses across sudangrass accessions. (A) Response to stem borer and bacterioses attacks. Accession *R*.*S 1842 Honey Sor*. was used as susceptibility control (B) Response to frost and drought. Accession *R*.*S 2305 I*.*S*. *1143 N*.*12* was used as susceptibility control.

Several phenological traits: flowering time, days to maturity, panicle exertion, number of tillers, plant height, productivity (Table 1) and response to frost, drought, bacterioses, and stem borer attack (Fig 5) were evaluated for phenotypic and genetic diversity of seven sudangrass accessions. Flowering time and maturity revealed a great variability among the genotypes studied, as shown by more than three weeks difference between the earliest and latest flowering and maturing genotypes, and in accordance with the statistically significant differences determined across accessions for days to flowering time (p <0.0001) and maturity (p <0.0001) (Table 1).

**Table 1:** Phenological traits across sudangrass accessions.

Sample name	Days to Flowering	Days to Maturity	Panicle exertion (cm)	Tiller number	Plant height (cm)^a^	Productivity (Kg DM / ha)^a^
***R*.*S 2199 Sudanense***	75±1.3 E	127±1.5 E	10±1.2 D	21±1.3 D	220±1.2 F	7354±44.3 F
***R*.*S 2198 Sudanense***	55±1.3 B	105±1.1 B	8±1.1 C	16±1.2 C	210±1.4 E	6981±52.9 E
***R*.*S 1370 Syn4***	52±2.0 A	105±1.7 B	5±0.7 B	13±1.7 B	115±1.0 A	3315±37.4 A
***R*.*S 841 Sudanense***	63±1.8 D	106±1.7 B	5±1.0 B	15±1.1 C	150±1.5 B	4683±57.5 B
***R*.*S 1601 Tift***	57±1.5 B	101±1.1 A	7±1.1 C	13±1.7 B	165±1.5 C	5263±58.0 C
***R*.*S 1594 Wealer***	60±1.9 C	112±2.3 D	3±0.7 A	9±1.6 A	150±1.3 B	4661±48.2 B
***R*.*S 1731 Juar 20***	62±1.7 C	110±1.3 C	10±1.3 D	15±1.3 C	200±1.8 D	6588±69.8 D

Means with the same letter are not significantly different (p> 0.05). (See S5 Table).

^a^ Plant height of sudangrass accessions was used to calculate productivity according to the regression between accumulated biomass (Kg DM/ha) and plant height (cm) [7].

Plant height also reflected significant variability (p <0.0001), and five groups of accessions with diverging plant height were clearly distinguished. The tallest genotype (220cm), *R*.*S 2199 Sudanense* almost double the plant height of *R*.*S 1370 Syn4*, the shortest genotype. Intermediate plant height groups, composed of *R*.*S 2198 Sudanense*, *R*.*S 841 Sudanense*, *R*.*S 1601 Tift*, *R*.*S 1594 Wealer*, *and R*.*S 1731 Juar 20* were 175cm tall, on average (Table 1).

**Fig 6:**
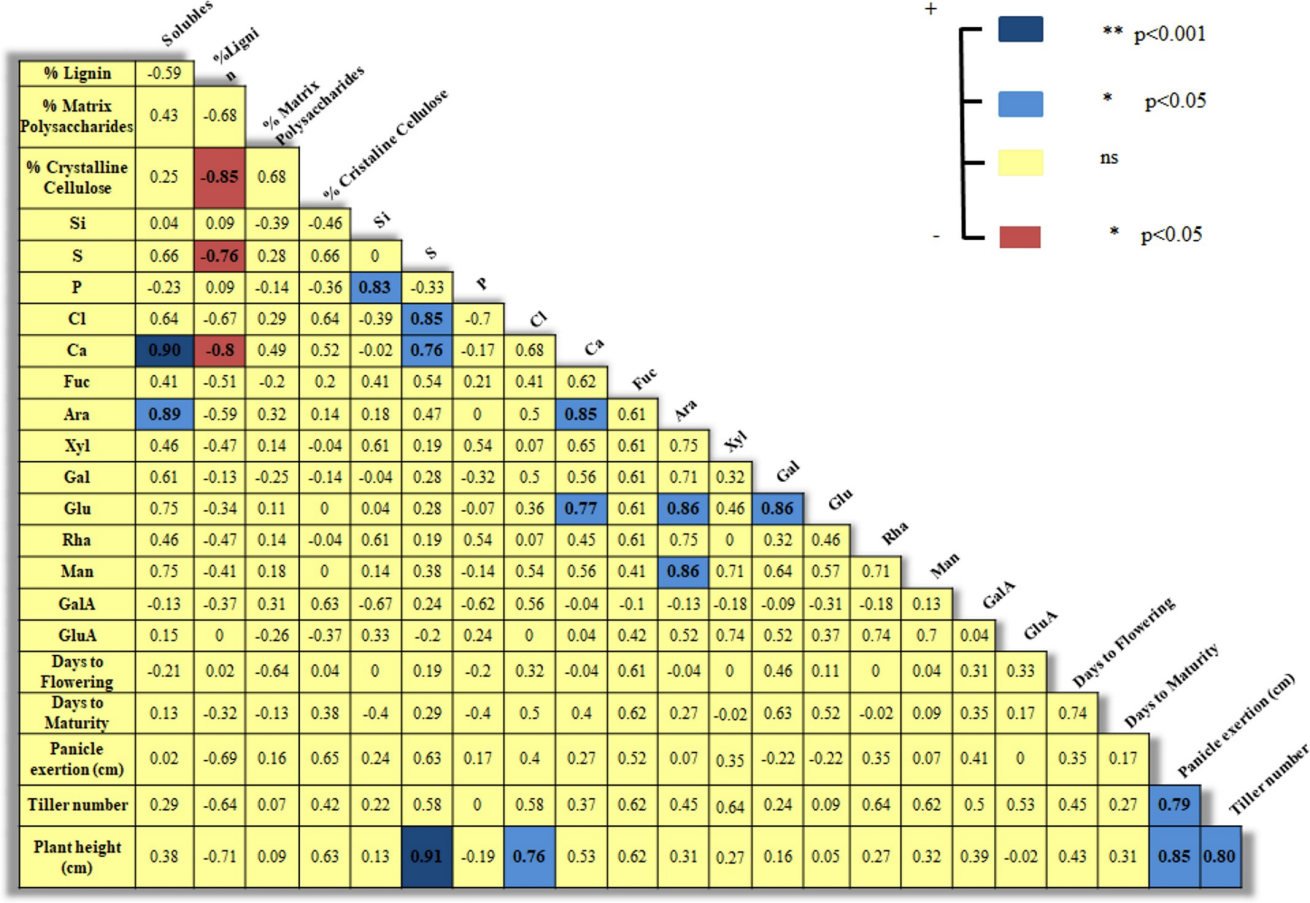
Spearman´s correlation coefficients among phenological traits, major polymers, monosaccharides, and elements in stem cell walls of sudangrass accessions.

Number of tillers differed significantly across accessions (p <0.0001). A positive correlation between number of tillers and plant height (r = 0.80; p = 0.0314*) was observed in the accessions studied (Fig 6). Panicle exertion, length of peduncle between the base of the panicle and the flag leaf of the plant, exhibited statistically significant differences across accessions (p <0.0001), and also correlated positively with plant height (r = 0.85; p = 0.0146*) and number of tillers (r = 0.79; p = 0.0357*) (Fig 6).

Sudangrass accession *R*.*S 1842 Honey Sor*., used as susceptible control for bacterioses and stem borer, scored 4 at 8-leaf developmental stage for bacterioses 3 at boot developmental stage for stem borer, indicating presence of bacterial inoculum and stem borer in the field (Fig 5A). Similar responses to both stresses had been observed on previous occasions under Manfredi environmental conditions, highlighting the relevance of using this accession as control to the named biotic stresses.

The response to stem borer was uniform across sudangrass accessions. Conversely, the response to bacterioses was more diverse, *R*.*S 1601 Tift* being the more tolerant genotype (Fig 5A).

Sudangrass accession *R*.*S 2305 I*.*S*. *1143 N*.*12*, used as susceptible control to drought and frost scored 4 to both stresses at flowering stage, in response to the lack of rainfall for almost three weeks and occasional minimum temperatures below the basal temperature of sorghum registered during this plant developmental stage (Fig 5B).

Response to biotic stresses reflected relevant variability among sudangrass genotypes (Fig 5B). As shown for bacterioses, *R*.*S 1601 Tift* also was the most tolerant accession to frost (Fig 5B). Two genotypes displayed the most tolerant response to drought: *R*.*S 1594 Wealer* and *R*.*S 841 Sudanense* (Fig 5B).

### Biomass composition and differential response to stresses

To investigate whether the content and composition of cell wall polymers relates with the response to abiotic stress and pathogen attack, we analyzed these parameters across different accessions.

Even though the content of soluble extractives showed statistically significant differences across sudangrass accessions, no relation was detected with the response to biotic and abiotic stresses (Figs 1A and 5). In a similar manner, the elemental composition of the different biomass samples showed no relation with the response to abiotic stress and pathogen attack (Figs 2 and 5).

*R*.*S 1731 Juar 20* accounted for the lowest concentration of Ara and scored 3 to bacterioses and 2 to frost, differing from *R*.*S 2199 Sudanense* that contained the highest concentration of Ara and scored 4 to bacterioses and 3 to frost (Figs 3 and 5). Likewise, *R*.*S 1731 Juar 20* accounted for the lowest concentration of Gal and scored 3 to bacterioses and 2 to frost, differing from *R*.*S 2199 Sudanense* that contained the highest concentration of Gal and scored 4 to bacterioses and 3 to frost (Figs 3 and 5). Also, *R*.*S 1594 Wealer* contained the lowest concentration of Xyl and scored 3 to bacterioses, 2 to frost and 1 to drought, differing from *R*.*S 2199 Sudanense* that contained the highest concentration of Xyl and scored 4 to bacterioses, 3 to frost and to 2 drought (Figs 3 and 5). Furthermore, *R*.*S 1601 Tift* accounted for the lowest concentration of GalA and scored 1 to bacterioses and frost, differing from *R*.*S 1731 Juar 20* that contained the highest concentration of GalA and scored 3 to bacterioses and 2 to frost (Figs 3 and 5).

Together, these findings suggest that accessions that exhibited the lowest concentrations of Ara, Gal, Xyl, and GalA appear to be accompanied by lower scores of response to bacterioses, frost, and drought than in accessions that exhibited the highest concentrations of these monossacharides. It is also likely, solely based on the monosaccharide composition that higher levels of Xyl and Ara in *R*.*S 2199 Sudanense* might stand for higher levels of arabinoxylans. The comparison of biotic and abiotic responses with saccharification analyses following sodium hydroxide pretreatment revealed higher amounts of reducing sugars in the most resistant genotypes to drought compared to the most susceptible one (Figs 4 and 5)

### Principal component analysis

**Fig 7:**
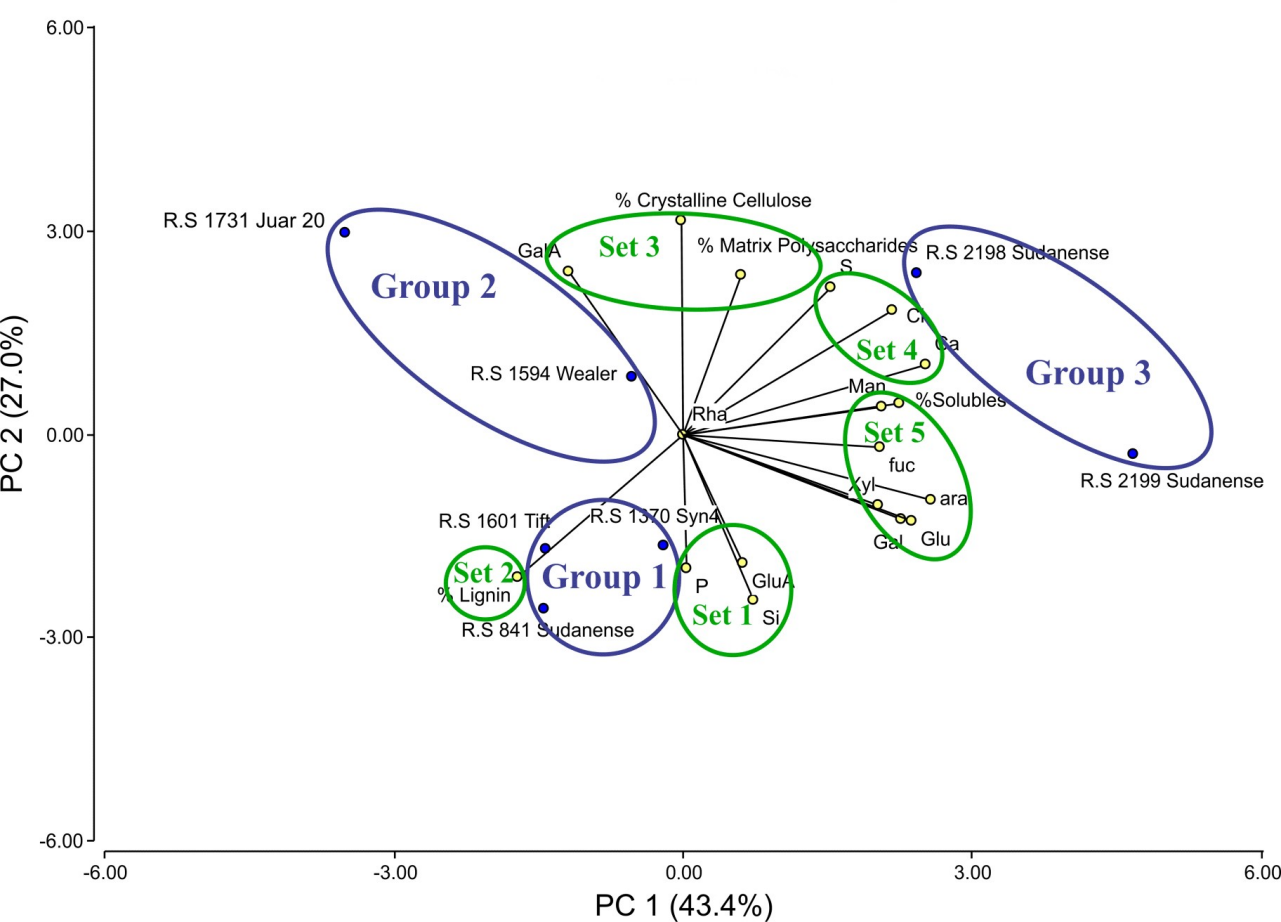
Principal component analysis for major cell wall polymers, monosaccharides, and agronomic parameters in seven sudangrass accessions. Sets (1, 2, 3, 4, and 5) of variables are encircled in green solid lines and groups (1, 2, and 3) of sudangrass accessions are encircled in blue solid lines.

The Principal component analysis (PCA) is a standard technique for visualizing high dimensional data, reducing the dimensionality (the number of variables) of a data set by maintaining as much variance as possible. For ease of interpretation, the full set of data from biomass and agronomic parameters, and cell wall elements and monosaccharides were subjected to PCA to obtain graphical representations of the relationships among the 7 sorghum genotypes (Fig 6). In the bi-plot, a summary of total variation of the analytical parameters is presented by the first (PC1) and second (PC2) principal components, which explained the 70.4% of total variance (Fig 7). PC1 and PC2 contributed to 43.4% and 27.0% of total variance, respectively, and distinguished three groups of genotypes (encircled in blue solid line) and five major sets of variables (encircled in green solid line). Group 1 is clearly defined and composed of accessions *R*.*S 1370 Syn4*, *R*.*S 841 Sudanense*, and *R*.*S 1601 Tift*; group 2 is composed of accessions *R*.*S 1731 Juar 20* and *R*.*S 1594 Wealer*; and group 3 of accessions *R*.*S 2198 Sudanense* and *R*.*S 2199 Sudanense*. Set 1 contained three variables: P, Si, and GluA; set 2 only contained lignin; set 3 contained GalA, crystalline cellulose, and matrix polysaccharides; set 4 contained S, Cl, and Ca; and set 5 contained seven variables: Man, solubles, Fuc, Ara, Xyl, Glu, and Gal (Fig 7)

The acute angles between vectors representing the variables of set 1 indicate that P, Si, and GluA were positively associated (Fig 7). Positive correlation between P and Si (r = 0.83; p = 0.0212*) is consistent with this result (Fig 6). A similar analysis can be applied to variables of set 3, 4 and 5. Within set 4, two positive correlations were determined: S with Cl (r = 0.85; p = 0.0153*) and Ca (r = 0.76; p = 0.0461*). Several positive correlations were detected within set 5: Ara with solubles (r = 0.89; p = 0.0287*), Glu (r = 0.86; p = 0.0358*), and Man (r = 0.86; p = 0.0358*); and between Gal and Glu (r = 0.86; p = 0.0358*) (Figs 6 and 7).

The sudangrass genotypes within group 1 were highly associated with lignin (Fig 7). *R*.*S 841 Sudanense and R*.*S 1601 Tift* displayed the highest and second highest values of lignin, respectively (Fig 1A). In its turn, lignin was negatively correlated with crystalline cellulose (r = -0.85; p = 0.0162*), S (r = -0.76; p = 0.0461*), and Ca (r = -0.80; p = 0.0307*) (Figs 6 and 7). Variables of set 3 were strongly associated with *R*.*S 1731 Juar 20* of group 2 (Fig 7), showing this genotype the highest value of crystalline cellulose and GalA (Figs 1A and 3B). As for Sorghum accessions of group 3 they were strongly associated with variables of set 4 since *R*.*S 2199 Sudanense* and *R*.*S 2198 Sudanense* exhibited the highest levels of S, Cl and Ca (Figs 7 and 1A).

## Discussion

Germplasm collections are important sources of genetic variability. Investigating the genetic, biochemical and agronomical features, and the bioenergy potential of the accessions comprising any germplasm collection underscores and justifies their conservation. *Sorghum sudanense* is a versatile species: the whole plant can be used in grazing, hay or silage. Identifying other uses beyond forage, would add alternatives to the agronomical systems where this crop is incorporated and provide extra income for farmers. To this end, seven sudangrass accessions, randomly chosen from a collection of sorghum germplasm, were analyzed to evaluate their potential as feedstocks for lignocellulosic bioethanol production, and to assess whether there is a correlation between the response to biotic and abiotic stresses and the composition of the biomass.

The complexity of the major structural and chemical components of secondary cell walls is the basis of lignocellulosic biomass recalcitrance [15]. Therefore, the analysis of the variability in biomass composition in accessions of *Sorghum sudanense* from a germplasm collection can provide insights into the potential for using this feedstock for bioenergy applications.

On average, the matrix polysaccharide and the crystalline cellulose fractions of sudangrass cell wall represented 68.16% of the total biomass, i.e. a potential source of sugars for bioethanol production bearing in mind that it can be converted into monosaccharides for fermentation [16]. The crystallinity of cellulose makes this polysaccharide difficult to hydrolyze by enzymes. Negative correlations between cellulose crystallinity and hydrolysis yield potential across different varieties of *Sorghum bicolor* have been reported [17]. Genetic engineering approaches aimed at reducing cellulose crystallinity in *Arabidopsis* [18] and increasing cellulose abundance in barley [19] by altering the expression of endogenous genes have been achieved with a significant penalty on plant growth and performance. As for *S*. *sudanense* it should be borne in mind that any attempt to reduce the crystalline cellulose fraction may lead to an increase of lignin content, in accordance with the negative correlation (r = -0.85; p = 0.0162*) estimated between crystalline cellulose and lignin (Fig 6) in the cell walls of the 5^th^ internodes of stems from sudangrass accessions.

In secondary cell walls, the polysaccharide network is impregnated and coated by lignin, providing rigidity and strength. On average, the lignin fraction represented 27.94% of sudangrass cell wall composition. Straw from rice lines with high reducing sugar release (above 90 nmol/mg. h) showed lower lignin content, suggesting that high saccharification potential in rice straw was due mainly to low lignin content [20]. Interestingly, rice lines with reducing sugar release below 90 nmol/mg.h showed more variation in lignin content, indicating that saccharification is controlled by multiple functional genes combined together to modulate the composition and interaction of the cell wall polymers [20].

Given the heterogeneous composition of lignocellulosic biomass, fermentation of pentose and hexose sugars derived from it represent a challenge. *S*. *cerevisiae* cannot ferment pentose sugars such as Xyl and Ara, although this drawback can be overcome by a range of metabolic engineering strategies [21, 22]. High concentrations of GalA in hydrolysates of pectin-rich feedstocks cannot be fermented either. These findings, together with the high pentose:hexose ratio (2.7:1) determined in sudangrass genotypes and the small amounts of GalA detected in the accessions (Fig 3), indicate that sudangrass biomass could be used for ethanolic fermentation. In this respect, the usage of the enriched-pentose accessions (*R*.*S 2199 Sudanense*, *R*.*S 2198 Sudanense*, and *R*.*S 1370 Syn4*) as feedstocks for alcoholic fermentation by engineered pentose-fermenting *S*. *cerevisiae* strains would be simplified and favored because these accessions contain low levels of GalA. In such case, the pretreatment should allow the recovery of the sugars derived from hemicelluloses.

The plant cell wall is a complex network of different polysaccharides that changes during plant development and in response to stress [23]. In the cell walls of the 5^th^ internodes of stems from sudangrass accessions the comparison of the composition in monosaccharides and arabinoxylans between susceptible and resistant genotypes to abiotic and biotic stresses exhibited differential responses. The most susceptible genotype to both frost and bacterioses (*R*.*S 2199 Sudanense*) had higher content of Ara, Xyl, Gal, Glu, and arabinoxylans in comparison to the most resistant ones (Figs 3 and 5), suggesting that the differences previously observed between genotypes might be mainly attributed to the hemicellulose polymers. This preliminary data is in accordance with the significantly lower percentages of Xyl and arabinoxylans detected in spikes of the *Fusarium* head blight resistant wheat 02-5B-318 in comparison with the sensitive genotype Saragolla; although, significantly higher percentage of Ara, Gal and Glu was determined in the resistant line compared to the susceptible one [24].

Several abiotic and biotic stresses have been associated with compositional changes in the cell wall. Arabinoxylan influenced disease resistance of barley against the powdery mildew fungus *Blumeria graminis* f. sp. *hordei* indicating that in monocots this hemicellulose is important in response to fungal infection [25]. The comparison of the cell wall in three wheat cultivars with different levels of tolerance to heat and drought exhibited an increase in arabinoxylan in all cultivars under both stress conditions [26].

Atkinson and Urwin (2012) [27] found evidence that plant responses to multiple environmental stresses is different from the response to individual stress factors. This is important considering that all field-grown sudangrass accessions were subject to multiple stresses. In this respect, a correlation between different cell wall characteristics and response to various environmental stresses needs to be examined in future studies. Cell wall analyses in stems of sudangrass accessions demonstrated significant variability in content of soluble extractives, monosaccharides, elemental composition, and stress responses, further enhancing the relevance of characterizing germplasm collections for bioenergy purposes.

## Supporting information

S1 Table Climatic data at Manfredi agricultural research station during the 2012–2013 campaign. Columns in the table show: Date, Mean Temperature at 150cm height, Maximum Temperature at 150cm height, Minimum Temperature at 150cm height, Rainfall, Mean humidity, Soil temperature at 10cm depth, Mean Wind speed at 200cm, and Mean Maximum wind speed.
(XLS)

S2 Table Major cell wall components in stems of sudangrass. Moisture ontent, ash content, solubles, matrix polysaccharides, lignin and crystalline cellulose were determined in different genotypes.
(XLSX)

S3 Table Monosaccharide composition in sudangrass genotypes. The amount of fucose (fuc), Arabinose (ara), Rhamnose (Rha), Galactose (Gal), Glucose (Glu), Xylose (Xyl), Manose (Man), Galacturoninc acid (GalA), snd Glucuronic acid (GluA) were determined using HAPAEC. For details, see Materials and Methods.
(XLSX)

S4 Table Saccharification analyses in sudangrass genotypes. Saccharification values are expressed in nmol of reducing sugars/mg material.hour.
(XLSX)

S5 Table Phenological traits across sudangrass accessions. Columns show Days to Flowering, Days to Maturity, Panicle exertion, Tiller number, Plant height, and Productivity across accessions.
(XLSX)

## References

[1] Bertello F. Averiado, pero en carrera: el campo pide pista para otra cosecha récord. La Nación. 29 sep 2018. Available from: https://www.lanacion.com.ar/2176619-averiado-pero-carrera-campo-pide-pista-otra.

[2] Reich JM. Utilizing the BMR trait in sudangrass and sorghums. In: Proceedings, California Alfalfa and Forage Symposium 2005 Dec; 12–14, Visalia, CA, UC Cooperative Extension, Agronomy Research and Extension Center, Plant Sciences Department, University of California, Davis 95616. Available from: http://alfalfa.ucdavis.edu

[3] Bibi A, Sadaqat HA, Akram HM, Khan TM, Usman BF. Physiological and agronomic responses of sudangrass to water stress. J. Agric. Res. 2010 1; 48(3): 369–380.

[4] Creamer NG, Baldwin KR. An evaluation of summer cover crops for use in vegetable production systems in North Carolina. HortScience 2000; 35(4): 600–603.

[5] Barhnhart S, Hartwig N. Managing immature or frost damaged sudangrass and sorghum crops. Iowa State University Extension. Recovery-27 9 1993; Ames, Iowa, USA.

[6] Rovira P, Echeverría J. Desempeño productivo de novillos pastoreando sudangras o sorgo forrajero nervadura marrón (BMR) durante el verano. Rev. vet. 2013; 24(2): 91–96

[7] Barbera P, Benítez J. Sorgo forrajero para pastoreo. INTA Ediciones. Colección Investigación, Desarrollo e Innovación. 2017; Serie Técnica Nº 5:16. ISBN 0327 / 3075.

[8] White GA, Clark TF, Craigmiles JP, Mitchell RL, Robinson RG, Whiteley EL, et al Agronomic and chemical evaluation of selected sorghums as sources of pulp. Economic Botany 1974 Apr-Jun; 28: 136–144.

[9] Oliver D, Thiselton-Dyer WT, Prain D, Hill AW. Sorghum sudanense (Piper) Stapf. D. Prain. London: L. Reeve and co. editor. In: Flora of Tropical Africa; 1917; 9(1): 113–114. https://www.biodiversitylibrary.org/item/231746#page/7/mode/1up

[10] Sorghum germplasm collection of INTA. Manfredi Research Experimental Station, Manfredi, Córdoba, Argentina. 2008.

[11] Foster CE, Martin TM, Pauly M. Comprehensive compositional analysis of plant cell walls (lignocellulosic biomass). Part I. 2010; Lignin. J. Vis. Exp. 37: e1745. 10.3791/1745 PMC3144576 20224547

[12] Jones L, Milne JL, Ashford D, McQueen-Mason SJ. Cell wall arabinan is essential for guard cell function. Proc. Natl. Acad. Sci. 2003; 100:11783–8. 10.1073/pnas.1832434100 13130074 PMC208835

[13] Gomez LD, Whitehead C, Barakate A, Halpin C, McQueen-Mason SJ. Automated saccharification assay for determination of digestibility in plant materials. 2010; Biotechnol. Biofuels. 3: 23–9. 10.1186/1754-6834-3-23 20979637 PMC2974669

[14] Di Rienzo JA, Casanoves F, Balzarini MG, Gonzalez L, Tablada M, Robledo CW. InfoStat versión 2017 Grupo InfoStat, FCA, Universidad Nacional de Córdoba, Argentina URL http://www.infostat.com.ar.

[15] Bhatia R, Gallagher JA, Gomez LD, Bosch M. Genetic engineering of grass cell wall polysaccharides for biorefining. Plant Biotechnol. 2017; 15: 1071–1092. 10.1111/pbi.12764 PMC5552484 28557198

[16] Marriott PE, Gómez LD, McQueen-Mason SJ. Unlocking the potential of lignocellulosic biomass through plant science. New Phytol. 2015; 209: 1366–1381. 10.1111/nph.13684 26443261

[17] Vandenbrink JP, Hilten RN, Das KC, Paterson AH, Feltus FA. Analysis of Crystallinity Index and Hydrolysis Rates in the Bioenergy Crop Sorghum bicolor. Bioenergy Res. 2012; 5(2): 387.

[18] Harris DM, Corbin K, Wang T, Gutierrez R, Bertolo, AL, Petti C, et al Cellulose microfibril crystallinity is reduced by mutating C-terminal transmembrane region residues CESA1A903V and CESA3T942I of cellulose synthase. Proc. Natl Acad. Sci. 2012; 109: 4098–4103. 10.1073/pnas.1200352109 22375033 PMC3306678

[19] Tan HT, Shirley NJ, Singh RR, Henderson M, Dhugga KS, Mayo GM, et al Powerful regulatory systems and post-transcriptional gene silencing resist increases in cellulose content in cell walls of barley. BMC Plant Biol. 2015; 15: 62 10.1186/s12870-015-0448-y 25850007 PMC4349714

[20] Liu B, Gómez LD, Hua C, Sun L, Ali I, Huang L, et al Linkage mapping of stem saccharification digestibility in rice. PLoS ONE. 2016; 11 (7): e0159117 10.1371/journal.pone.0159117 27415441 PMC4944936

[21] Kötter P, Ciriacy M. Xylose fermentation by *Saccharomyces cerevisiae*. Appl. Microbiol. Biotechnol. 1993; 38: 776–783.

[22] Wisselink HW, Toirkens MJ, Franco Berriel MR, Winkler AA, van Dijken JP, Pronk JT, et al Engineering of *Saccharomyces cerevisiae* for efficient anaerobic alcoholic fermentation of L-arabinose. Appl. Environ. Microbiol. 2007; 73: 4881–4891. 10.1128/AEM.00177-07 17545317 PMC1951023

[23] Scheller HV, Ulvskov P. Hemicelluloses. Annu. Rev. Plant Biol. 2010; 61:263–89. 10.1146/annurev-arplant-042809-112315 20192742

[24] Lionetti V, Giancaspro A, Fabri E, Reem N, Bellincampi D. Cell wall traits as potential resources to improve resistance of durum wheat against Fusarium graminearum. BMC Plant Biol. 2015; 15:6 10.1186/s12870-014-0369-1 25597920 PMC4298115

[25] Chowdhury J, Henderson M, Schweizer P, Burton RA, Fincher GB, Little A. Differential accumulation of callose, arabinoxylan and cellulose in nonpenetrated versus penetrated papillae on leaves of barley infected with *Blumeria graminis* f. sp. *hordei*. New Phytol. 2014; 204:650–660. 10.1111/nph.12974 25138067

[26] Domon JM, Baldwin L, Acket S, Caudeville E, Arnoult S, Zub H, et al Cell wall compositional modifications of Miscanthus eco- types in response to cold acclimation. Phytochem. 2013; 85: 51–61. 10.1016/j.phytochem.2012.09.001 23079767

[27] Atkinson NJ, Urwin PE. The interaction of plant biotic and abiotic stresses: from genes to the field. J. Exp. Bot. 2012 6; 63(10): 3523–3543. 10.1093/jxb/ers100 22467407

